# Cardiovascular magnetic resonance assessment of the aortic valve stenosis: an *in vivo* and *ex vivo* study

**DOI:** 10.1186/s12880-015-0076-x

**Published:** 2015-08-26

**Authors:** Stefan Buchner, Kurt Debl, Franz-Xaver Schmid, Andreas Luchner, Behrus Djavidani

**Affiliations:** Klinik und Poliklinik für Innere Medizin II, Universitätsklinikum Regensburg, Franz-Josef-Strauss-Allee 11, 93042 Regensburg, Germany; Institut für Röntgendiagnostik, Universitätsklinikum Regensburg, Regensburg, Germany; Klinik und Poliklinik für Herz-, Thorax- und herznahe Gefäßchirurgie, Universitätsklinikum Regensburg, Regensburg, Germany

**Keywords:** Aortic valve, Aortic stenosis, Magnetic resonance imaging, Anatomy

## Abstract

**Background:**

Aortic valve area (AVA) estimation in patients with aortic stenosis may be obtained using several methods. This study was undertaken to verify the cardiovascular magnetic resonance (CMR) planimetry of aortic stenosis by comparing the findings with invasive catheterization, transthoracic (TTE) as well as tranesophageal echocardiography (TEE) and anatomic CMR examination of autopsy specimens.

**Methods:**

Our study was performed in eight patients with aortic valve stenosis. Aortic stenosis was determined by TTE and TEE as well as catheterization and CMR. Especially, after aortic valve replacement, the explanted aortic valves were examined again with CMR *ex vivo* model.

**Results:**

The mean AVA determined in vivo by CMR was 0.75 ± 0.09 cm^2^ and ex vivo by CMR was 0.65 ± 0.09 cm^2^ and was closely correlated (r = 0.91, *p* < 0.001). The mean absolute difference between AVA derived by CMR *ex vivo* and *in vivo* was −0.10 ± 0.04 cm^2^. The mean AVA using TTE was 0.69 ± 0.07 with a significant correlation between CMR ex vivo (r = 0.85, *p* < 0.007) and CMR *in vivo* (r = 0.86, *p* < 0.008). CMR *ex vivo* and *in vivo* had no significant correlation with AVA using Gorlin formula by invasive catheterization or using planimetry by TEE.

**Conclusion:**

In this small study using an ex vivo aortic valve stenosis model, the aortic valve area can be reliably planimetered by CMR *in vivo* and *ex vivo* with a well correlation between geometric AVA by CMR and the effective AVA calculated by TTE.

## Background

Aortic valve stenosis is the most common cardiac valve disease resulting in valve therapy [1]. Exact determination of the severity of stenosis is essential to guide therapy [2]. Standard methods, such as cardiac catheterization and transthoracic echocardiography (TTE) have to calculate the effective orifice area by measurement of the transvalvular pressure gradient [3, 4], as they do not allow a direct and precise measurement of the geometric orifice area. Therefore it seems desirable to directly determine the geometric orifice area by a flow independent technique such as planimetry. Several cardiologists take the view that TTE using the Doppler-technique is now the best reference (“gold standard”) whereas the Gorlin-formula is the historical reference.

Cardiovascular magnetic resonance (CMR) is a noninvasive method that allows visualization of cardiac function, structure and valves [5, 6]. Recently, we and others have reported the success of planimetry by CMR in aortic valve [7–14].

However, to date, no studies have been published describing *ex vivo* CMR stenotic aortic valves. It therefore seemed useful to compare anatomic data with CMR imaging.

We hypothesized that direct planimetry of the stenotic aortic valve in an autopsy human valve as a standard of reference using CMR corresponds to the planimetry of the stenotic aortic valve *in vivo* using CMR. To address this hypothesis, we performed a head-to-head comparison between aortic valve area (AVA) planimetry preoperatively by CMR and postoperatively of the explanted aortic valve by CMR in a series of eight patients with aortic valve stenosis. In addition, CMR measurements were compared with TTE and transesophageal echocardiography (TEE) as well as catheterization data

## Methods

### Patients

Eight patients referred to our institution for evaluation of aortic valve stenosis and were prospectively included in the study undergoing aortic valve replacement. All diagnostic procedures and measurements were performed by experienced observers, who were blinded to the results of the other imaging modalities. Informed consent was obtained in all patients. The study approved by local ethics committee of the Universität Regensburg.

### Clinical characteristics of the patients

Clinical data of the eight patients are summarized in Table 1. Mean age was 76 ± 6 (62 to 82) years. The majority of patients presented with clinical symptoms of severe aortic stenosis, such as systolic murmur, dyspnea, chest pain or syncope. Concomitant aortic regurgitation was present in 5 patients and was defined as mild/trivial (*n* = 4) or moderate (*n* = 1) by CMR and echocardiography. TEE planimetry of AVA was not possible in one patient because of heavy calcifications. Planimetry of AVA was possible in all patients undergoing CMR. An impaired image quality was observed in one patient due to trigger problems by cardiac arrhythmia.

**Table 1:** Patient characteristics

Patient	Age	Sex	Rhythm	HR	SBP	NYHA	CCS	Syncope
1	80	female	SR	71	130	4	0	0
2	82	female	AF	77	158	3	1	0
3	74	female	SR	91	125	2	0	0
4	75	female	SR	67	115	3	0	1
5	79	female	SR	83	120	3	2	0
6	75	male	AF	97	116	4	3	0
7	62	female	SR	63	130	3	4	0
8	77	female	SR	80	130	3	2	0

SR, sinus rhythm; AF, atrial fibrillation; HR, heart rate; SBP, systolic blood pressure; NYHA, New York Heart Association, CCS, Canadian Cardiovascular Society

### Transthoracic echocardiography

Standardized transthoracic echocardiography was performed with measurements of the left ventricular outflow tract diameter, the velocity of across the aortic valve and flow data of the left ventricular outflow tract to calculate the AVA using the continuity equation [15].

### Transesophageal Echocardiography

Multiplane TEE was performed using a 5-Mhz annular phased array probe. For examination of the aortic valve, the imaging plane was rotated from 0° to 180° until the best image of the aortic valve opening in the short axis view was obtained (usually around 60°). Minimal probe manipulation was performed to ensure that the smallest orifice of the aortic valve at the leaflet tips was identified. Planimetry of the smallest orifice at the time of maximum opening in early systole (triggered by the electrocardiogram) was performed. At least three consecutive measurements were averaged.

### Catheterization

A standard cardiac catheterization procedure was performed via the percutaneous femoral approach, including right and left heart pressure measurement. Peak to peak and mean pressure gradients were determined between left ventricle and ascending aorta. Cardiac output was measured by thermodilution, averaging at least 3 measurements. AVA was estimated using the Gorlin formula [16].

### Surgery and autopsy

The description of the aortic valve was obtained from one surgeon. We obtained human valves from patients with aortic valve stenosis at the time of surgical valve replacement. Despite the heavy calcification, all valves could be explanted totally. Specimens were originally preserved in formaldehyde solution.

### Cardiovascular magnetic resonance studies – *in vivo*

All patient studies were performed on a 1.5 T scanner (Sonata, Siemens Medical Solutions, Erlangen, Germany). CMR studies were performed in supine position with a phased-array receiver coil and breath-hold acquisitions prospectively gated to the ECG. Cine images were acquired in multiple short axis and long axis views with fast imaging with steady state free precession (trueFISP, slice thickness 8 mm, echo time 1.53 ms, readout bandwidth 1.085 Hz/pixel, repetition time 3.14 ms, matrix 256*202).

Image analysis was performed off-line using the semiautomatic ARGUS evaluation program (Siemens Medical Solutions, Erlangen, Germany), which is a part of the commercially available cardiac package of the scanner software.

The imaging plane of the aortic valve was defined by acquiring a systolic 5-chamber view parallel to the long axis of the left ventricular outflow tract and a long axis view of the left ventricular outflow tract and the proximal aorta, perpendicular to the 5-chamber view, as described previously [7]. In brief, the subsequent slices were defined parallel to the valvular plane and, additionally, in cases of orifices with an eccentric outlet, perpendicularly to the direction of the jet. At least 4 slices (range 4–7) at different levels of the aortic valve were acquired and the imaging plane with the smallest orifice was chosen. Planimetry of the smallest orifice at the time of maximum opening in early systole in the acquisitions prospectively triggered to the electrocardiogram was performed by two independent observers, who were unaware of the echocardiographic, catheterization and ex vivo results. We placed our traces at the point of the bright pixels. Three measurements were performed and average for calculating the AVA. Intraobserver and interobserver variabilities regarding AVA were 3 % and 6 % for *in vivo* CMR, respectively.

### Cardiovascular magnetic resonance studies – *ex vivo*

**Fig. 1:**
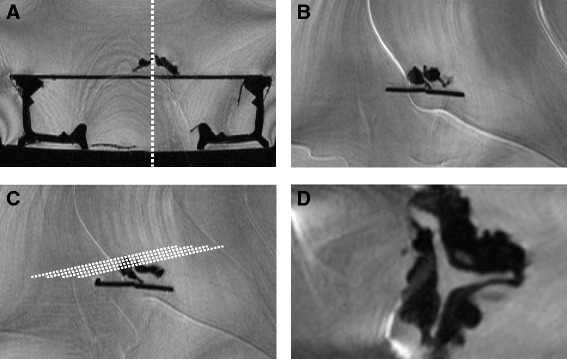
Position of the *ex vivo* aortic valve fixed in a water container and planning the slices. **a**, overview; **b**, coronar view; **c**, angulation for planning the orthograd view; **d**, the resulting orthograd view

**Fig. 2:**
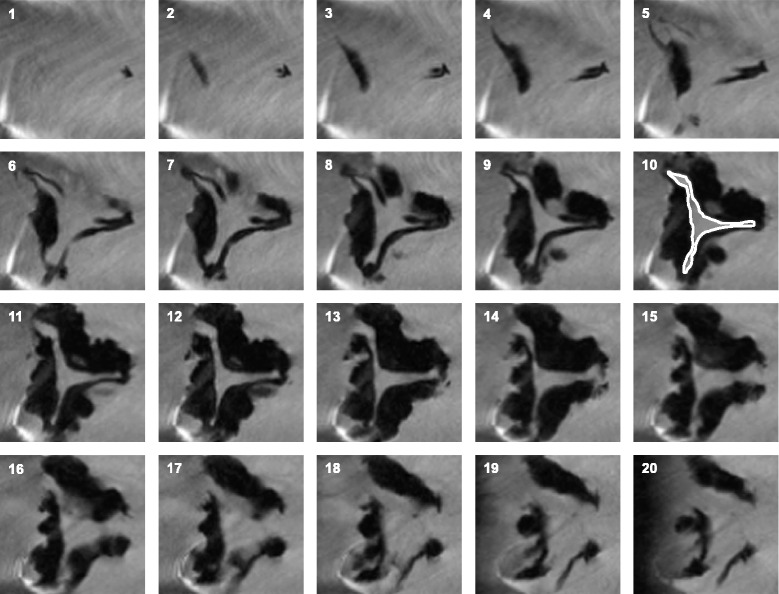
To avoid assessing the aortic valve area beyond or above the leaflet tips, the imaging plane was moved shift wise in one mm steps in an orthograd direction. Planimetry was chosen on the slice where the smallest orifice was surrounded totally by the edge of the valve

All specimens were examined on a 1.5 T scanner (Magnetom Sonata, Siemens Medical Solutions, Erlangen, Germany). The CMR imaging protocol included a 3D-CISS protocol (TR/TE/flip angle 17/8.08 ms/70° ms, band width 130 Hz/pixel, effective slice thickness 1 mm pixel size 0.6 × 0.45 mm). The aortic valve was placed on a rack into a water container. Then the container with the aortic valve was placed in the gantry. Slices in the orthogonal planes (transverse, coronal and sagittal) were obtained (Fig. 1). Serial angulated-axis views from the base to tip of the valve were acquired with 3D sequence (Fig. 2). Planimetry of the smallest orifice was performed by two independent observers, who were unaware of the echocardiographic, catheterization and in vivo results. We placed our traces at the edge of the bright pixels. Three measurements were performed and average for calculating the AVA. Intraobserver and interobserver variabilities were 1 % and 4 % for *ex vivo* CMR, respectively.

**Fig. 3:**
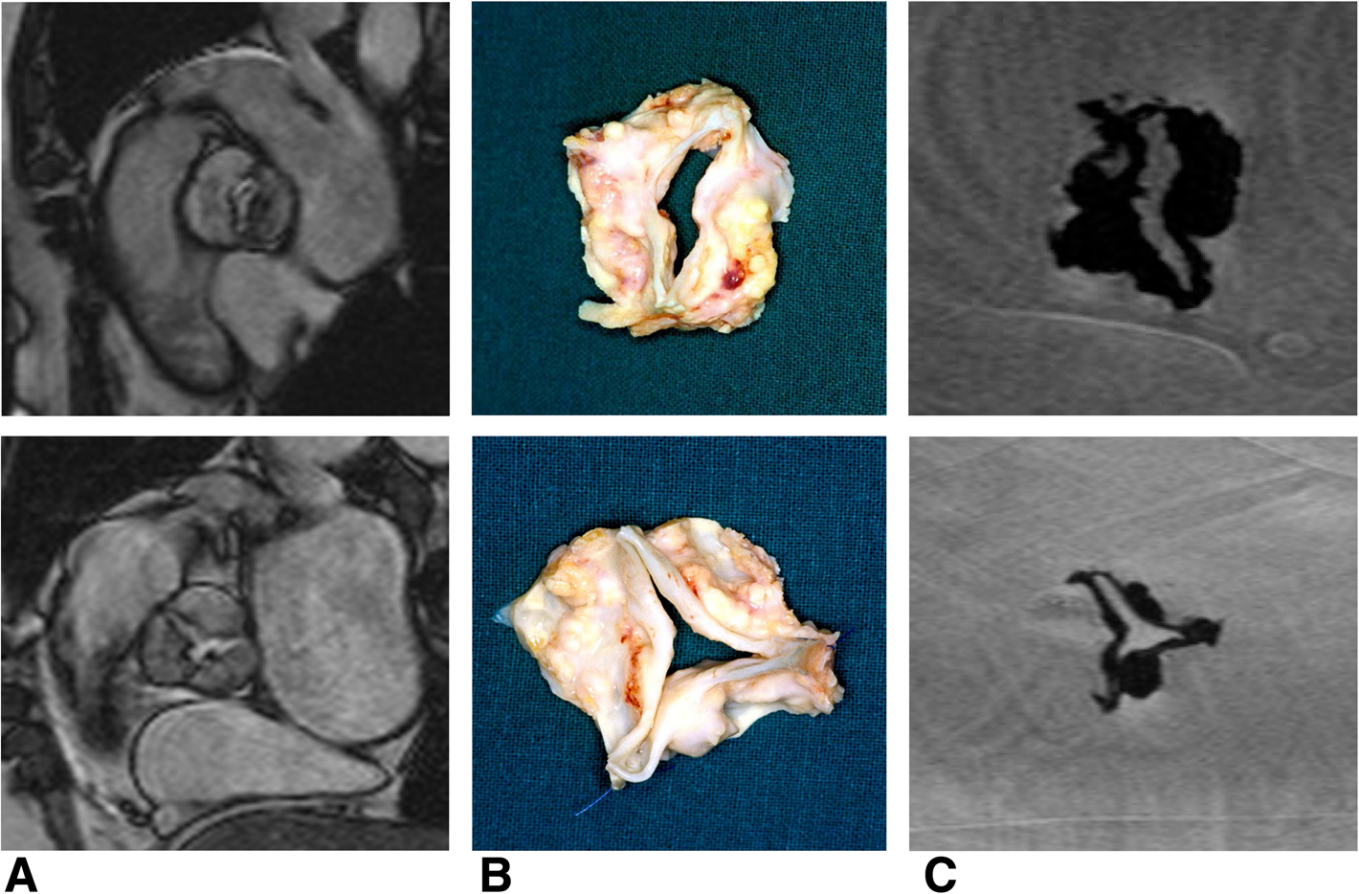
Images derived from the in vivo CMR (**a**), from the aortic valve specimen (**b**) and from the ex vivo CMR (**c**) in a bicuspid stenotic valve (top) and in a tricuspid stenotic valve (bottom)

### Statistical analysis

Data expressed as mean ± standard deviation. Linear regression analysis was performed to describe correlations between the different techniques. The mean AVA of the different techniques was compared by student's t-Test for paired samples. Intra- and interobserver variability (*n* = 8) were expressed as the percentage of variability (absolute value of the difference between 2 measurements divided by the mean of 2 measurements). Agreement between the different techniques was assessed as described by Bland and Altman. A level of significance of below 0.05 was defined as statistically significant. SPSS version 21 (SPSS Institute, Chicago) was used for statistical analysis.

## Results

### CMR planimetry of AVA *in vivo* and *ex vivo*

The measurements of all patients by the various modalities are depicted in Table 2. Six valves were tricuspid and two valves were bicuspid. Images of aortic valve specimen, in vivo and ex vivo CMR are depicted in Fig 3.

**Table 2:** Results

Patient	Aortic valve type CMR	Aortic valve type surgery	AVA *in vivo* by CMR (cm^2^)	AVA *ex vivo* by CMR (cm^2^)	AVA by TEE (cm^2^)	AVA by TTE (cm^2^)	AVA by CATH (cm^2^)	PG mean by Doppler (mmHg)	PG mean by CATH (mmHg)	LV EF by CMR (%)	LV Mass by CMR (g)	LV SV by CMR (ml)
1	BAV	BAV	0.84	0.76	-	0.74	0.29	65	77	50	151	61
2	TAV	TAV	0.70	0.55	0.65	0.65	0.36	59	65	41	126	36
3	TAV	TAV	0.84	0.73	0.70	0.75	0.64	53	45	81	177	81
4	TAV	TAV	0.86	0.70	0.80	0.75	0.52	76	76	59	178	73
5	TAV	TAV	0.62	0.50	0.70	0.55	0.34	44	46	19	134	40
6	BAV	BAV	0.76	0.69	1.10	0.65	0.85	40	35	45	210	72
7	TAV	TAV	0.72	0.68	0.75	0.73	0.67	49	55	77	113	57
8	TAV	TAV	0.71	0.59	0.80	0.69	1.08	30	20	71	120	50

AVA, aortic valve area; BAV, bicuspid aortic valve; CMR, cardiovascular magnetic resonance; CATH, cardiac catheterization; TEE, transesophageal echocardiography; TTE, transthoracic echocardiography; LV EF, left ventricular ejection fraction; LV mass, left ventricular mass; LV SV, left ventricular stroke volume; PG, pressure gradient; TAV, tricuspid aortic valve

**Fig. 4:**
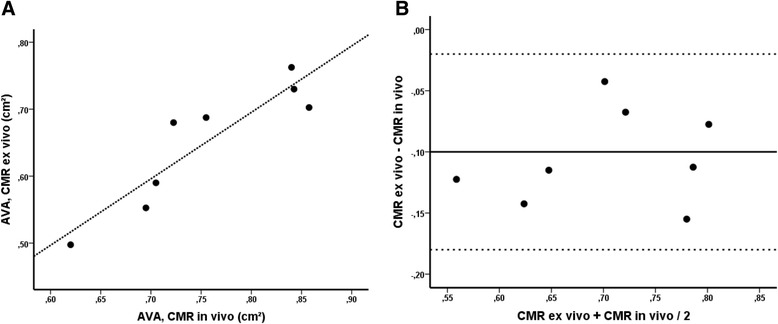
Agreement between aortic valve area assessed by CMR in vivo and by CMR ex vivo (**a**). According to Bland-Altman (**b**), the difference between the two comparative measurements is plotted against their mean. The continuous line represents the mean difference and dashed lines represent the limits of agreement (mean difference ± 2SD)

The mean AVA determined *ex vivo* by CMR was 0.65 ± 0.09 cm^2^. Mean AVA determined *in vivo* by CMR was 0.75 ± 0.09 cm^2^. The mean absolute difference between AVA derived by CMR *ex vivo* and *in vivo* was 0.10 ± 0.04 cm^2^ (*p* < 0.001, Fig. 4 and Table 3 ). The AVA *in vivo* and *ex vivo* by CMR was closely correlated (r = 0.91, *p* < 0.001).

The mean absolute difference between AVA derived by CMR ex vivo and calculated using the continuity equation by TTE was −0.04 ± 0.05 cm^2^ (*p* = 0.066). The mean absolute difference between AVA derived by CMR *in vivo* and calculated using the continuity equation by TTE was +0.07 ± 0.04 cm^2^. In linear regression analysis the correlation between CMR ex vivo and TTE (r = 0.85, *p* < 0.007) was similar to that between CMR *in vivo* and TTE (r = 0.86, *p *< 0.008).

The mean absolute difference between aortic valve area derived by CMR ex vivo and TEE was +0.12 ± 0.09 cm^2^ (*p* = 0.012). The mean absolute difference between aortic valve area derived by CMR *in vivo* and TEE was −0.02 ± 0.10 cm^2^ (*p* = 0.75). In linear regression analysis the correlation between CMR ex vivo and TEE was r = 0.44, *p* = 0.323) and between CMR *in vivo* and TEE r = 0.28, p = 0.551.

The mean absolute difference between AVA derived by CMR ex vivo and calculated using the Gorlin formula by catheterization was +0.07 ± 0.26 cm^2^ (*p* = 0.498). The mean absolute difference between AVA derived by CMR *in vivo* and calculated using the Gorlin formula by catheterization was +0.17 ± 0.27 cm^2^ (*p* = 0.117). In linear regression analysis the correlation between CMR *ex vivo* and Gorlin formula by catheterization was r = 0.09, *p* = 0.835 and between CMR *in vivo* and Gorlin formula by catheterization was r = −0.03, *p* = 0.952.

### AVA with other methods

The mean of the aortic valve area calculated using the Gorlin formula by catheterization was 0.60 ± 0.27 cm^2^. The mean aortic valve area determined by TEE was 0.79 ± 0.15 cm^2^. The mean of the aortic valve area calculated by TTE was 0.69 ± 0.07 (Table 3).

**Table 3:** Comparison of methods of planimetry and calculation of aortic valve area

	Aortic valve area (cm^2^)	
Method	Mean	Range
CMR *ex vivo*	0.65 ± 0.09	0.50 – 0.76
CMR i*n vivo*	0.75 ± 0.09	0.62 – 0.86
TEE	0.80 ± 0.16	0.65 – 1.10
TTE	0.69 ± 0.07	0.50 – 0.70
CATH	0.59 ± 0.27	0.29 – 1.08

CMR, cardiovascular magnetic resonance; CATH, cardiac catheterization; TEE, transesophageal echocardiography; TTE, transthoracic echocardiography

The mean absolute difference between AVA derived calculated using the Gorlin formula by catheterization and calculated using the continuity equation by TTE was −0.11 ± 0.25 cm^2^ (*p* = 0.278). The mean absolute difference between AVA using the Gorlin formula by catheterization and TEE was −0.13 ± 0.19 cm^2^ (*p* = 0.122). The mean absolute difference between AVA calculated using the continuity equation by TTE and TEE was −0.08 ± 0.10 cm^2^ (*p* = 0.97). In linear regression analysis the correlation between Gorlin formula by catheterization and TEE was r = 0.71, *p* = 0.073 and between Gorlin formula by catheterization and the continuity equation by TTE was r = 0.17, *p* = 0.639 and between TEE and catheterization and the continuity equation by TTE was r = 0.15, *p* = 0.748.

## Discussion

The results of this study demonstrate that aortic valve stenosis can be accurately planimetered by using CMR *in vivo* compared with ex vivo model. Precise assessment of aortic valve area in aortic stenosis is crucial for optimal patient treatment [17]. However, no comparison of planimetry of the aortic valve area has made with *in vivo* and *ex vivo* aortic valves by CMR or other modality. The present study is the first to validate the ability of CMR to accurately and precisely planimetry aortic valve stenosis in vivo comparing by *ex vivo* CMR.

Characterization of the severity of aortic stenosis is among the most difficult problems in valvular heart disease. Exact determination of the severity of stenosis is essential to guide therapy [18]. In evaluating the severity of the aortic valve stenosis, it is ideally desirable to determine geometric orifice area On the other hand, the opening of the aortic valve is though highly dependent of the flow and gradient. Because planimetry only provides geometric orifice area and does not characterize the flow property, this could be a challenge in low-flow/low-gradient aortic stenosis [19–21]. The guidelines allow various methods to determine the AVA. However, the different available techniques often might lead to discrepancy results. This could demonstrate in the current study and previous studies which show that the planimtery of the AVA was systematically higher than the calculated AVA by Gorlin in catheterization or continuity equation in TTE. This is due to the fact that direct planimetry reflects the anatomical orifice while the calculated valve area reflects the effective orifice area. Despite the different available techniques for the assessment of the AVA all available techniques have their limitations and this should be keep in mind. In TTE, the inexact measurement of the diameter of the left ventricular outflow tract or missed peak transvalvular velocity might lead to a false AVA. In TEE, heavily calcification of the aortic valve could lead to impaired image quality e.g. by the acoustic shadowing, thus exact delineation of the cusps and planimetry of the AVA are impossible or the potential inability to identify the accurate imaging plane for planimetry. Hemodynamic assessment of the aortic valve area by invasive cardiac catheterization has often been challenged because of potential imprecision introduced by the hemodynamic parameters. Finally, CMR has its limitation in valve motion and exact slice orientation or in contraindication for CMR (e.g. claustrophia or metallic implants).

Prior to our study, several other investigators have determined the accuracy of several methods for CMR and cardiac computed tomography planimetry of the aortic valve using an angiographic reference standard or Doppler derived data [7–9, 22, 23]. However, the ultimate test of the value of the method seems to be comparison with anatomy. The current study extends these findings as it demonstrates that CMR allows to accurately visualizing the anatomic valve area *in vivo* compared to *ex vivo*.

To date, most CMR studies for the quantification of stenotic aortic valves have focused on direct planimetry [24], although the dimensions of CMR voxels relative to the size and shape of stenotic orifices sometimes influence this approach. Therefore, an *ex vivo *study was deemed as an essential intermediate study for assessing sources of errors in CMR aortic valve estimation prior to *in vivo* experiments. Ex vivo estimates eliminate errors due to motion blurring, which includes errors resulting from irregular heart rates and images acquired during motion. Furthermore, ex vivo determinations also minimize uncertainties due to edge definition and produced high-contrast edges in which the aortic valve boundary is defined to be pixel values above background. The results of this study should, therefore, be interpreted as the minimum errors which can be achieved using CMR.

Despite the close correlation between *ex vivo* and *in vivo* planimetry by CMR, we observed an underestimation of the aortic valve area by CMR *ex vivo*. The underlying reason for underestimation of the aortic valve area by CMR may be related to valve motion and slice orientation. Specifically, transplanar valve motion during systole might lead to overestimation of valve area when the imaging plane misses the smallest orifice area. Taking measurement from CMR, one has to make sure that the plane is orthogonal and on the edge of the leaflets. *Ex vivo* imaging, however, eliminates potential errors caused by cardiac and respiratory motion.

### Limitations

Several limitations of our study should be noted. The sample size of the current study was small. Most of the patients had been diagnosed with high-grade aortic valve stenosis, resulting in a selection bias. However, because we decided to use combined left and right heart catheterization and study the explanted aortic valve, we were unable to include patients with mild aortic stenosis, because, in these patients these would not be justified. Furthermore, only the static area of the aortic valve was measured and do not reflect the hemodynamic conditions *in vivo*. However, it is difficult to assess the dynamic change of stenotic aortic valve area in ex vivo specimens. In this study only due to the heavy calcification of the aortic valve, it was possible to explant the valve as a unit and we presume that the shape of the valve represent the maximum opening of the valve in systole. Consequently, the aortic valve area of the explanted aortic valve corresponds by the calcification the smallest maximal orifice may merely influenced by ejection and diastolic valve closing.

## Conclusion

In conclusion, this is the first study to validate planimetry in aortic valve stenosis using CMR in an *ex vivo* model. CMR *in vivo* slightly overestimates the valve area in aortic stenosis compared to CMR ex vivo. Furthermore, planimetry of AVA using CMR *in vivo* and *ex vivo* model correlated well with calculated AVA by TTE, in contrast to TEE and invasive measurements of AVA. These certain methodical discrepancies between these methods must be taken into account when grading the severity of aortic stenosis by AVA.

## Abbreviations

AVA: Aortic valve area
CMR: Cardiac magnetic resonance
TEE: Transoseophageal echocardiography
